# PGC-1α Protects RPE Cells of the Aging Retina against Oxidative Stress-Induced Degeneration through the Regulation of Senescence and Mitochondrial Quality Control. The Significance for AMD Pathogenesis

**DOI:** 10.3390/ijms19082317

**Published:** 2018-08-07

**Authors:** Kai Kaarniranta, Jakub Kajdanek, Jan Morawiec, Elzbieta Pawlowska, Janusz Blasiak

**Affiliations:** 1 Department of Ophthalmology, University of Eastern Finland, 70211 Kuopio, Finland; kai.kaarniranta@kuh.fi; 2 Department of Ophthalmology, Kuopio University Hospital, 70029 Kuopio, Finland; 3 Department of Molecular Genetics, Faculty of Biology and Environmental Protection, University of Lodz, Pomorska 141/143, 90-236 Lodz, Poland; jakub.kajdanek@unilodz.eu; 4 Department of General and Colorectal Surgery, Medical University of Lodz, Pl. Hallera 1, 90-647 Lodz, Poland; jmorawiec@op.pl; 5 Department of Orthodontics, Medical University of Lodz, Pomorska 251, 92-216 Lodz, Poland; elzbieta.pawlowska@umed.lodz.pl

**Keywords:** PGC-1α, age-related macular degeneration, AMD, senescence, mitochondrial quality control, mitochondria, oxidative stress

## Abstract

PGC-1α (peroxisome proliferator-activated receptor gamma coactivator 1-alpha) is a transcriptional coactivator of many genes involved in energy management and mitochondrial biogenesis. PGC-1α expression is associated with cellular senescence, organismal aging, and many age-related diseases, including AMD (age-related macular degeneration), an important global issue concerning vision loss. We and others have developed a model of AMD pathogenesis, in which stress-induced senescence of retinal pigment epithelium (RPE) cells leads to AMD-related pathological changes. PGC-1α can decrease oxidative stress, a key factor of AMD pathogenesis related to senescence, through upregulation of antioxidant enzymes and DNA damage response. PGC-1α is an important regulator of VEGF (vascular endothelial growth factor), which is targeted in the therapy of wet AMD, the most devastating form of AMD. Dysfunction of mitochondria induces cellular senescence associated with AMD pathogenesis. PGC-1α can improve mitochondrial biogenesis and negatively regulate senescence, although this function of PGC-1α in AMD needs further studies. Post-translational modifications of PGC-1α by AMPK (AMP kinase) and SIRT1 (sirtuin 1) are crucial for its activation and important in AMD pathogenesis.

## 1. Introduction

Age-related macular degeneration (AMD) is a complex disease occurring in two clinically distinguished forms: dry (non-exudative) and wet (exudative). It is a major cause of legal blindness in the elderly in developed countries, and despite that millions of AMD patients lose their sight each year, there is no effective treatment for dry AMD [1]. AMD is associated with several risk factors, and many of them are linked to increased oxidative stress. On the other hand, oxidative stress can be involved in several mechanisms of AMD pathogenesis [2]. The stress is characterized by an excess of reactive oxygen species (ROS), which are produced mainly in mitochondria, even in their normal functioning. Therefore, regulation of mitochondrial function can be critical for maintaining ROS at physiological levels, and its impairment can be linked to the pathogenesis of many diseases, including AMD [3]. This is supported by reports on decreased mitochondrial mass, disturbed mitochondrial network, and diminished expression of mitochondrial proteins in AMD patients [4,5,6]. AMD primarily affects retinal pigment epithelium (RPE) cells with the subsequent degeneration of photoreceptors. PGC-1α (PPARGC1A, peroxisome proliferator-activated receptor gamma coactivator 1-alpha) is a protein involved in the regulation of many cellular aspects, including mitochondrial homeostasis and ROS level control [7]. Therefore, it can be an important player in AMD pathogenesis. The knockout mouse model for the PGC-1α gene has been used to mimic human AMD [8]. Moreover, PGC-1α can be implicated in other mechanisms important in AMD etiology, including autophagy. PGC-1α can also play an important role in the senescence of RPE cells in oxidative stress, and it was postulated that senescence of RPE cells is a critical event in the degeneration of the retina occurring in AMD [9,10]. Therefore, there are many reasons and rationales to study the role of PGC-1α in AMD pathogenesis.

Oxidative stress and ROS excess in AMD cannot be entirely attributed to dysfunctional mitochondria, as it was shown that accumulation of all-trans-retinal (atRAL), an important intermediate of the visual cycle, led to NADPH (reduced nicotinamide adenine dinucleotide phosphate) activation, resulting in ROS production and apoptosis of RPE cells [11]. That work also showed that atRAL excess induced nitrosative stress that can also contribute to AMD pathogenesis. However, it was shown that during apoptosis of RPE cells, accumulation of a free form of atRAL in ARPE-19 cells led to mitochondrial dysfunction, DNA damage, and endoplasmic reticulum stress [12,13,14]. Therefore, atRAL can play an important role in AMD pathogenesis, and its action can be underlined by oxidative stress, which can be potentiated by mitochondria impairment, but it is not completely clear whether dysfunctions in atRAL clearance belong to the reasons or consequences of AMD, or both.

## 2. PGC-1α—The Gene and the Protein

The peroxisome proliferator-activated receptor γ coactivator 1 (PGC-1) proteins are master regulators of energy production and expedition in the cell [7]. Their expression is expected in tissues with a high energy need: brown adipose tissue, heart, brain, liver and skeletal muscle, and PGC-1α and PGC-1β belong to the most important energy regulators in these tissues. Therefore, it is not surprising that most studies on PGC-1α were performed in skeletal muscle cells [15].

The PGC-1α gene, PPARGC1A, has been mapped to the 4p15.2 cytogenetic band and is 717, 731 bp (http://www.genecards.org/cgi-bin/carddisp.pl?gene=PPARGC1A&keywords=pgc-1a). Two independent promoters, canonical and alternative, have been identified in that gene [16,17]. Initially, 13 exons and 12 introns were localized in the human PPARGC1A gene, with exon 1 containing the translation initiation codon [18]. The TAA stop codon and two polyadenylation signals (AATAAAs), as well as the 3′ UTR (untranslated region), were found in 3′ part of the gene. The PPARGC1A gene has 18 transcripts, resulting from alternative splicing of its pre-mRNAs, occurring mainly in intron 6 [19]. Additionally, an alternative first exon has been identified in mouse [16]. This exon splices to the canonical exon 2, and constitutes a part of the 5′ UTR and codons of an alternative mRNA [17].

**Figure 1:**
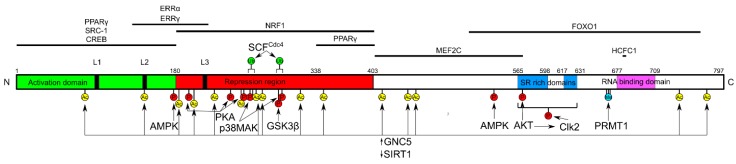
Domain structure of the PGC-1α (peroxisome proliferator-activated receptor gamma coactivator 1-alpha) protein. Numbers represent amino acid positions. L1-L3—leucine-rich domains, SR—serine and arginine-rich domains. PGC-1α interacts with transcription factors in a domain-specific fashion (horizontal lines above) and is post-translationally phosphorylated (P), acetylated (Ac), methylated (Me), and ubiquitinated (Ub) (mostly below). GNC5 acetylates and SIRT1 deacetylates PGC-1α. Full names of proteins interacting with PGC-1α are in the main text.

PGC-1α has a complex, multidomain structure, associated with its key role in the regulation of various cellular processes, resulting from co-activation of nuclear receptors (NRs) and transcription factors (TFs) (Figure 1) [20].

PGC-1α has three N-terminal leucine-rich domains, L1-L3, and two of them, L2 and L3, interact with the ligand-binding domain of NRs. The repression region of PGC-1α is bound by nuclear respiratory factor 1 (NRF-1), which is a transactivator of genes essential for mitochondrial biogenesis. C-terminal and central domains of PGC-1α participate in the interaction with myocyte-specific enhancer factor 2C (MEF2C), peroxisome proliferator-activated receptor γ (PPARγ), forkhead fox O1 (FOXO1), and host cell factor C1 (HCFC1). All domains of PGC-1α can be involved in its interaction with TFs, assisting the recruitment of other transcriptional co-activators, including the Mediator complex, estrogen-related receptor α and γ (ERRα and ERRγ), CREB (cAMP response element)-binding protein, and Src proto-oncogene, non-receptor tyrosine kinase-1 (SRC1) [7,21,22]. Post-translational modifications are an important element of the regulation of the PGC-1α gene expression and transcriptional activity of its protein, and are mainly expressed by phosphorylation by AMP kinase (AMPK), p38 MAPK (mitogen-activated protein kinase), AKT (protein kinase B), Clk2 (CDC like kinase 2, and GSK-3β (glycogen synthase kinase 3β)). However, acetylation by GNC5 (general control nonderepressible 5) and ubiquitination by SCFCdc4 (Skp1/Cullin/F-box Cell division control protein 4) play an important role in PGC-1α activity, as this protein is normally in an inactive state, underlined by its acetylation in multiple sites [23,24,25,26]. For full activation, PGC-1α needs to be deacetylated by SIRT1 (sirtuin 1) that is the only protein able to do so, and phosphorylated by AMPK [27]. Also, GlcNAcylation of PGC-1α is a mechanism of its post-translational modification [28]. It seems that the activity of PGC-1α is regulated mainly at two levels: transcriptional and post-translational [29]. The former is associated with the amount of the protein, and may represent a slow, adaptive response to a moderate factor, whereas the latter represents the way to respond to a sudden influence. This two-level regulation of PGC-1α activity can serve its precise control.

PGC-1α regulates different physiological processes in different tissues, but transcriptional control of cellular respiration and mitochondrial biogenesis occurs in all sites [30].

## 3. PGC-1α—A Key Regulator of Mitochondrial Biogenesis and Redox Control

The integration of processes to maintain mitochondrial homeostasis results in a collective mechanism of mitochondrial quality control (mtQC). These processes occur within the organelle, and include turnover of mtDNA, heme, and protein, but also comprise effects affecting whole mitochondria, such as mitophagy—a selective mitochondrial autophagy, and mitochondrial biogenesis [31].

NRF-1 is a nuclear transcription factor, and an essential regulator of mitochondrial biogenesis and function [32,33]. It is involved in the synthesis of proteins important for the transport through the mitochondrial membrane and proteins of the respiratory chain. Moreover, it activates the expression of mitochondrial transcription factors TFAM (mitochondrial transcription factor A), TFBM1, and TFBM2 (mitochondrial transcription factor B1 and B2), which increase the transcription of mtDNA, and stimulate the activity of mitochondrial polymerase RNA, as well as mtDNA replication [34]. However, NRF-1 needs co-activation to effectively act in mitochondrial biogenesis, which is provided by members of the PGC-1 protein family, including PGC-1α [35,36,37]. As mentioned, to be fully activated, PGC-1α must be deacetylated and phosphorylated. Oxidative stress can result in mitochondrial dysfunction, leading to increased AMP/ATP ratio and NAD+ level. This, in turn, will activate AMPK and SIRT1, which can direct PGC-1α activation to increase the expression of mitochondrial genes to protect against oxidative stress and produce energy.

**Figure 2:**
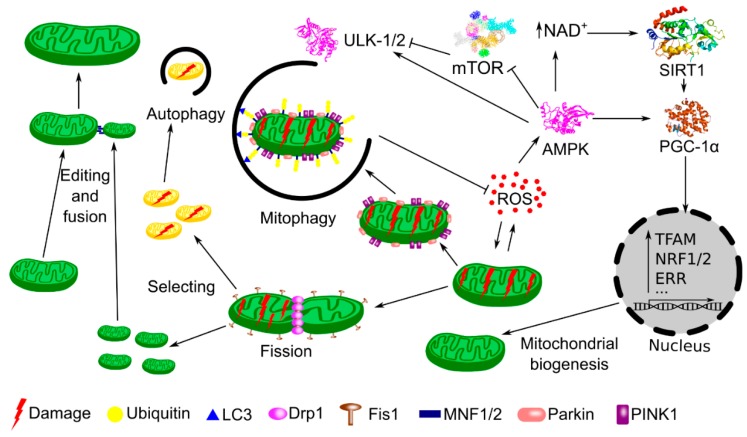
PGC-1α (peroxisome proliferator-activated receptor gamma coactivator 1α) is a master regulator of mitochondrial quality control (mtQC) during stress. Increased concentration of reactive oxygen species (ROS) results in damage to mitochondria. Damaged mitochondria are sensed by the joint action of PINK1 and Parkin. Dynamin related protein 1 (Drp1) and mitochondrial fission 1 protein (Fis1) are involved in dissection of damaged mitochondria (fission) into fragments, which can be ubiquitinated and degraded (autophagy, mitophagy), with the involvement of microtubule-associated protein 1A/1B-light chain 3 (LC3), or repaired (edited) and included into dynamic mitochondrial network (fusion) with the involvement of mitofusin 1 and 2 (MNF1/2). However, damaged mitochondria may accelerate ROS production, which activates pathways to prevent detrimental consequences of oxidative stress. Increased ROS activate AMPK (5′ AMP-activated protein kinase), which phosphorylates PGC-1α and increases NAD+ (nicotinamide adenine dinucleotide) concentration, leading to the activation of SIRT1 (Sirtuin 1). Concerted action of AMPK and SIRT1 results in PGC-1α phosphorylation and deacetylation, respectively, necessary for its activation. PGC-1α transactivates many genes encoding proteins essential in mitochondrial biogenesis, such as TFAM (mitochondrial transcription factor A), NRF-1 and 2 (nuclear respiratory factor 1 and 2), ERRs (estrogen-related receptors), and others (···). In mitophagy, AMPK inactivates mTOR (mammalian target of rapamycin), which inhibits ULK1 and 2 (Unc-51 like autophagy activating kinase 1 and 2). Mitochondria, through fission and fusion, are also able to repair damaged components by segregating or exchanging material (editing process).

Oxidative stress and other detrimental conditions in the cell can lead to mitochondrial damage. The Ser/Thr kinase PINK1 (PTEN (phosphatase and tensin homolog)-induced putative kinase 1) and the E3 ubiquitin ligase Parkin act in concert to sense a damaged mitochondria and direct it to degradation in the mitophagy pathway [38]. Damaged mitochondria are fragmented (mitochondrial fission) and fragments, which no longer maintain mitochondrial potential, are removed via mitophagy (Figure 2) [39]. Remaining fragments can be fused (fusion), repaired (edition) and mtDNA replication and gene expression can be induced, leading to a biogenic repair of the mitochondrial network. PGC-1α was shown to increase the transcription of proteins involved in the mitochondrial fusion, including mitofusin 1 and 2 [40,41]. In the context of general properties of PGC-1α, it is interesting that AMPK is partly responsible for mitophagy induction, and contributes to PGC-1α activation to initiate mitochondrial biogenesis [42].

PGC-1α activates uncoupling protein-1 and -2 (UCP-1, UCP-2), that are anion carriers, and acts protectively in mitochondria by reducing oxidative stress [43,44,45]. Additionally, UCP-2 is expressed in ganglionar cells, so its mitochondrial-related action could have a neuroprotective character in the retina [46]. Borniquel et al. suggested that nitric oxide can interact with PGC-1α to regulate, both positively and negatively, the mitochondrial antioxidant defense system in the vascular endothelium [47]. The authors observed a protein kinase G (PKG)-mediated downregulation of PGC-1α.

It was shown that the expression of PGC-1α increased mitochondrial respiration in cells obtained from patients with mitochondrial diseases underlined by deficiency in the complex III or IV resulted from mutations in the nuclear and mitochondrial DNA [48].

## 4. Stress-Induced Senescence and Mitochondrial Dysfunction—Major Factors of AMD Pathogenesis

Cellular senescence is an emerging element of loss of fitness associated with aging and age-related diseases, such as AMD. Senescence can be considered as a hallmark of organismal aging, so it can be, directly or indirectly, related to AMD, in which aging is, per definition, a main factor of pathogenesis. Senescent cells are irreversible non-replicating, and they display mitochondrial and telomere dysfunctions and elevated oxidative stress [49].

**Figure 3:**
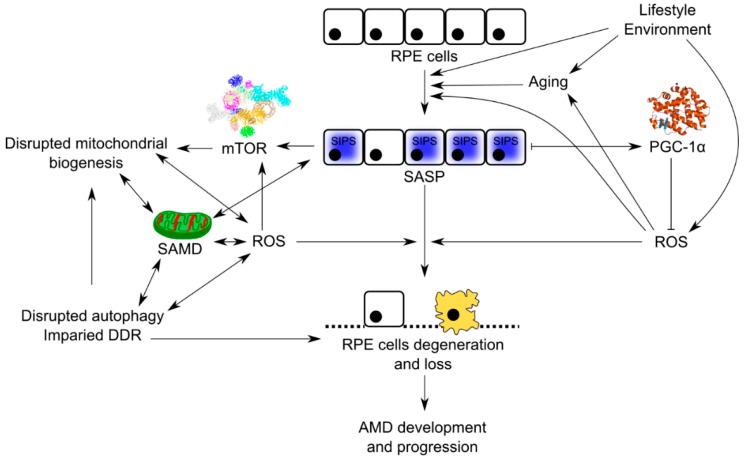
Senescence may significantly contribute to the pathogenesis of age-related macular degeneration (AMD). Peroxisome proliferator-activated receptor gamma coactivator 1α (PGC-1α) can decrease AMD-related detrimental changes. Retinal pigment epithelium (RPE) cells in the aging retina are continuously exposed to many environmental and lifestyle stress factors that accelerate aging and damage of RPE cells, leading to their senescence and lack of ability to regenerate their damaged and degenerated counterparts. Overproduction of ROS is an important element in this pathway as it is coupled with impaired DNA damage reaction (DDR) and dysfunctional autophagy, important elements of AMD pathogenesis, also contributing to senescence of RPE cells, which show senescence-associated secretory phenotype (SASP) and senescence-associated mitochondrial dysfunction (SAMD). Damage to RPE cells leads to degradation of organelles, including mitochondria, via mTOR (mechanistic target for rapamycin kinase)-dependent autophagy/mitophagy. PGC-1α can decrease ROS levels and protect against ROS-induced effects, including disrupted mitochondrial biogenesis.

Senescent cells produce more ROS, which damage mitochondria and genes crucial for mitochondrial homeostasis [50]. Many stress factors can induce premature cellular senescence (SIPS, stress-induced premature senescence), resulting in senescence-associated secretory phenotype (SASP) and senescence-associated mitochondrial dysfunction (SAMD). SAMD can stimulate accelerated aging, which is associated with several serious disorders. Cell senescence can be a driving force of age-related tissue dysfunctions and pathologies, partly related to SASP [51,52,53]. Both SASP and ROS can induce and stabilize senescence in neighboring cells. It was suggested that senescence, and not programmed cell death, is critical for the degeneration of RPE in AMD, and we have developed this suggestion and built a model of AMD pathogenesis with senescence as its critical element (Figure 3) [9,10]. Impaired autophagy and DNA damage response in RPE cells interplay with senescence in this model of AMD pathogenesis.

The central RPE contains cells that are quiescent due to spatial constraints, and contact with the neural retina. However, once damaged, they can activate an endogenous compensatory mechanism, leading to their replacement with proliferating RPE cells from the peripheral regions of RPE [54]. This mechanism is stimulated in stress conditions and potentiated by aging [55]. If most of the RPE cells, including in the periphery, are affected by senescence, this mechanism fails, resulting in a massive degeneration of central RPE cells, typical of AMD. SIPS is often driven by an excessive oxidation, and the mature human retina is characterized by one of the highest—if not the highest—oxygen use among all human tissues [56].

As mentioned, cellular senescence and mitochondrial dysfunction are mutually dependable, but have been independently identified as essential markers and causes of aging [57]. Further studies are needed to establish the exact, mechanistic role of this complex relationship in aging and age-related diseases. Many reports indicate an important role of mitochondrial impairment in AMD pathogenesis [58,59,60,61,62,63]. There are many aspects of this role, including variability in mtDNA associated with altered bioenergetics, increased mtDNA damage, fewer and smaller mitochondria, and their displaced localization in RPE cells and others, pointing at mitochondria as a major cellular structure involved in AMD pathogenesis. However, it is still to be determined whether all these effects belong to the reasons of the disease, or are its consequences.

RPE cells perform several functions essential for phototransduction. One of them is the degradation of photoreceptors outer segments (POS) by phagocytosis. POS are stacked phospholipid membrane discs, which are internalized by RPE cells with the involvement of CD36 (cluster of differentiation 36) and MerTK (Mer tyrosine kinase), initiating their degradation by the autophagic/lysosomal pathway [64,65,66,67]. Non-degraded POS and other material can accumulate in RPE cells as lipofuscin, which can lead to their senescence [68,69]. However, cultured RPE cells treated with POS showed a decreased sensitivity to oxidative stress and resulting apoptosis, suggesting that POS can be important in RPE cells physiology, protecting them from senescence [70]. Moreover, lack of active POS phagocytosis in mice was associated with accelerated age-related retinal dysfunction [71]. Therefore, there must be a delicate balance between POS important for RPE cell physiology and non-degraded POS, determining sensitivity to oxidative stress, senescence, and aging in RPE cells, all important in AMD pathogenesis. As PGC-1α can be involved in protection against oxidative stress, regulating senescence and, in these ways, influence aging, it can contribute to this balance.

Korolchuk et al. have presented convincing arguments that senescence results in impaired mitophagy leading to SAMD [50]. SAMD, in turn, can contribute to accelerated aging. Therefore, as aging is a major risk factor for AMD, and cellular senescence can be important in etiology of this disease, impaired mitophagy and SAMD can play an important role in AMD pathogenesis.

## 5. Involvement of PGC-1α in Senescence Regulation in RPE Cells

SIPS can be related to increased levels of ROS and ROS-related DNA-damage signaling. Therefore, cellular antioxidant defense, including antioxidant enzymes, DNA repair proteins, and small-molecular weight antioxidants, protects against SIPS. It was shown that PGC-1α was involved in antioxidant defense through interaction with Nfe212 (nuclear factor erythroid 2-related factor 2), a transcription factor important in protection against oxidative stress resulting from damage and inflammation [33]. That study also showed that superoxide dismutase 2 (SOD2), an enzyme clearing an excess of mitochondrial ROS, was activated by PGC-1α.

**Figure 4:**
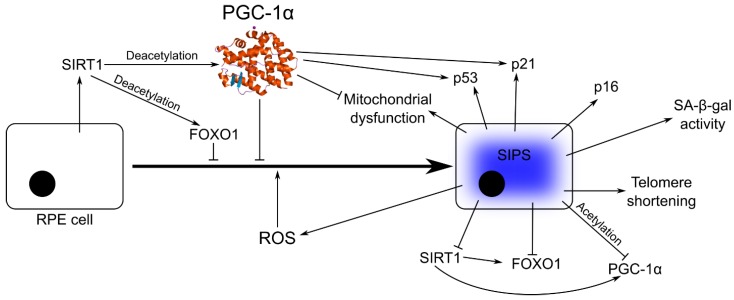
PGC-1α is a negative regulator of senescence in RPE cells. Oxidative stress underlined by an ROS excess leads to stress-induced premature senescence (SIPS), characterized by increased expression of SA-β-gal, p53, p21, p16, which can be modulated by PGC-1α. SIPS is also associated with mitochondrial dysfunction, which can be improved by PGC-1α and telomere shortening, inducing DNA damage response with a major involvement of p53, stimulated by PGC-1α. Increased expression of SIRT1 induces deacetylation of FOXO1 and PGC-1α, resulting in senescence attenuation. Full names of proteins are in the main text.

Senescent cells show increased expression of senescence-associated β-galactosidase (SA-β-gal), tumor suppressor protein p53, and cyclin-dependent kinase inhibitors, including p21 (CIP1 (CDK (cyclin-dependent kinase)-interaction protein 1)/WAF1 (wild-type p53-activated fragment 1)) and p16 [72,73]. Replicative senescence is associated with telomere shortening to the critical length (crisis), and further shortening usually leads to cell death [74]. This crisis can be overcome by the activation or increased expression of telomerase or alternative mechanisms of telomere lengthening. However, telomere dysfunction occurs not only in replicative senescence, but in SIPS as well. Increased expression of the SIRT1 deacetylase was observed to attenuate cellular senescence, through an increased deacetylation of FOXO1 [75]. However, SIRT1-mediated deacetylation is also needed for full activation of PGC-1α. Therefore, PGC-1α can be a negative regulator of senescence (Figure 4). Despite indispensable function of PGC-1α in redox control and mitochondrial biogenesis, few studies were performed on the role of this protein in the retina, which is the organ of the highest energy demand and highest oxygen flow. Moreover, the molecular mechanisms of coupling of energy expedition with the repair of damaged mitochondria are completely unknown. It was shown that PGC-1α was expressed at high levels in the mouse retina with a maximum in photoreceptors [8]. Mice with deleted PGC-1α and exposed to high-intensity light showed impaired retinal functions and disturbance in the expression of genes involved in retinal apoptosis and repair. In vitro overexpression of PGC-1α resulted in a strong anti-apoptotic effect. This important work has shown that, besides managing of energy production, PGC-1α is involved in fundamental processes of retina repair, renewal, and regeneration, confirming an essential role of PGC-1α in tissue homeostasis.

In an excellent study, Xiong et al. showed that PGC-1α deficiency in single-knockout mice resulted in senescence in vascular cells, with signs typical of replicative senescence and mitochondrial dysfunction [76]. In addition, they observed a reduced expression of SIRT1, telomerase, and catalase (CAT), an enzyme catalyzing decomposition of hydrogen peroxide to water and oxygen, and increased expression of p53. It should be stressed that even reduction of CAT activity can be sufficient to induce senescence, and its overexpression in mitochondria was associated with prolonged longevity in mice [77,78,79]. To search for the mechanisms of observed effects, Xiong et al. noted that hormonal induction of senescence resulted in acetylation of PGC-1α and clearing of the SIRT1 gene promoter from PGC-1α/FoxO1 complex, resulting in decreased expression of SIRT1. PGC-1α acetylation resulted in downregulation of SIRT1 and catalase, and a decrease in senescence underlined by disrupted interaction among PGC-1α, FOXO1, and SIRT1. They finally concluded that PGC-1α is a principal negative regulator of senescence. Although those experiments were performed on mouse vascular and rat aortic smooth muscle cells, they indicate PGC-1α as a main player in senescence regulation and pathogenesis of age-related chronic diseases.

Senescence of RPE cells can be linked with changes in PGC-1α functioning. As mentioned, PGC-1α is important in antioxidant defense, and its malfunction can contribute to increased ROS levels, inducing senescence and SASP. However, it was shown that a decreased level of ROS was not associated with milder consequences of senescence [80]. Moreover, PGC-1α was shown to regulate lysosomal activity in RPE cells by the TFEB (transcription factor EB) protein, which might improve autophagy flux and removal of cell damage [81,82]. It has been also demonstrated that PGC-1α-deficient mice developed some abnormalities in RPE cells that were associated with their accelerated senescence. Next, PGC-1α silencing in RPE cells aggravated senescence induced by hydrogen peroxide. These cells showed a significantly stronger and more frequent SA-β-gal staining, a hallmark of SASP, compared to control cells. It was shown that hydroxytyrosol, an antioxidant, exerted its protective action against oxidative stress by the stimulation of mitochondrial biogenesis involving PGC-1α activation [83]. PGC-1α in RPE cells can induce antioxidant enzymes, including superoxide dismutase (SOD), catalase (CAT), and glutathione peroxidases (GPX) [8]. PGC-1α was shown to be involved in maturation of the human RPE cells, and its overexpression was associated with overexpression of oxidative phosphorylation and fatty acid oxidation genes [84]. Increased expression of PGC-1α was also associated with induction of several antioxidant genes and protected RPE cells from oxidant-related death without impairing their basal functions.

**Figure 5:**
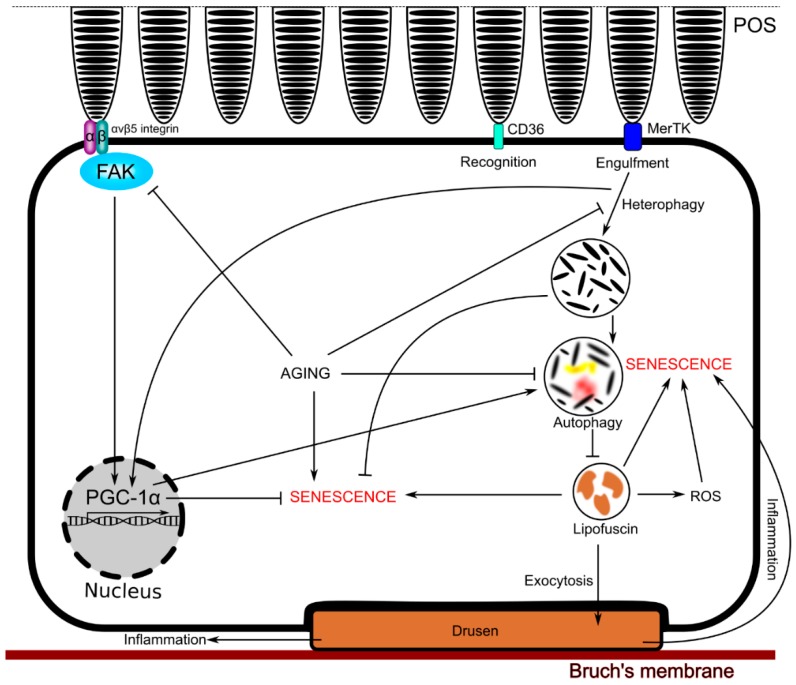
Phagocytosis of photoreceptor outer segments (POS) in RPE cells can contribute to the protective action of PGC-1α against AMD. POS are internalized by RPE cells with the involvement of CD36 and MerTK, initiating their degradation by the autophagic/lysosomal pathway. Non-degraded POS and other material accumulate as lipofuscin, contributing to senescence. However, low levels of POS can protect RPE cells from senescence, and lack of POS phagocytosis results in accelerated age-related retinal dysfunctions. RPE cells bind POS by the activation of the avb5 αvβ5 integrin/FAK (focal adhesion kinase)/PGC-1α pathway. Full names of proteins are in the main text.

As mentioned, removal of POS by phagocytosis in RPE cells is essential for correct phototransduction, so it plays a physiological role. Ueta et al. showed that phagocytosis of POS in undifferentiated ARPE-19 cells was associated with an increased expression of PGC-1α [85]. Therefore, as phagocytosed POS can play a role in anti-senescent action of PGC-1α in RPE cells, POS phagocytosis in RPE cells can contribute to the mechanisms of protective action of PGC-1α against AMD induction and development. However, the mechanism of this protection is not completely known. To search for this mechanism, Roggia and Ueta showed that the binding of POS by RPE cells was associated with the activation of the αvβ5 integrin/FAK (focal adhesion kinase)/PGC-1α pathway [81]. They also showed that this activation facilitated POS lysosomal degradation, declined with age, and resulted in protection against oxidative stress in RPE cells (Figure 5).

## 6. Potential of PGC-1α in AMD

AMD is a neurodegenerative, chronic disease with a strong connotation to aging. Lowered expression of PGC-1α has been established to play a role in the occurrence and progression of other age-related neurodegenerative disorders, including Huntington, Parkinson, and Alzheimer diseases [86,87,88]. This, at least in part, justifies the consideration of the protective role of PGC-1α in AMD.

Wet AMD is featured by choroidal neovascularization, caused by neoangiogenesis with an important involvement of VEGF (vascular endothelial growth factor), whose overexpression in RPE cells is observed in such forms of AMD. The introduction of anti-VEGF substances in the treatment of wet AMD was a breakthrough in the therapy of this disease [89]. Ueta et al. showed that intense, but physiological light upregulated VEGF, leading to choroidal neovascularization and this effect was mediated by PGC-1α [85]. More precisely, they showed that a stimulation with intense light enhanced POS phagocytosis by RPE cells, which induced the activation of the PGC-1α/ERRα pathway, which, in turn, upregulated VEGF. Therefore, targeting PGC-1α can be considered in anti-VEGF strategies to increase their efficacy in wet AMD treatment.

PGC-1α can cooperate with p53 in fighting oxidative stress and its consequences in the form of dysfunction of telomeres and mitochondria [90]. This mechanism suggests the involvement of DNA damage response (DDR) in the protective action of PGC-1α against age-related diseases. This was confirmed by Xiong et al., who showed that PGC-1α deletion increased DNA damage in vascular smooth muscle cells [91]. DNA damage was evaluated by the comet assay, and the production of 8-OH-dG (8-hydroxydeoxyguanosine), which is considered as a hallmark of oxidative DNA damage and oxidative stress in general. We and others have shown that AMD patients display an impaired DDR, evidenced by enhanced levels of damage to nuclear and mitochondrial DNA and decreased efficacy of DNA repair [9,60,92,93]. However, these studies did not unequivocally show a mechanism of observed changes. Although, in some cases, impaired DDR was associated with specific variants of DDR genes, it is not sure whether DDR alterations in AMD belong to reasons or consequences of the disease [94]. In the context of the potential role of PGC-1α in AMD pathophysiology, studies on its relation to DDR in AMD are justified and needed. These studies should also include senescence, as DNA damage is a prototype of cell cycle arrest, a hallmark of senescence. Moreover, the crucial role of PGC-1α in mtQC and the accumulation of oxidative DNA damage with aging suggest a self-feeding loop, in which DNA damage, induced by oxidative stress, results in perturbations in the mitochondrial electron transport chain increasing the stress, which stimulates SIRT1 to deacetylate and activate PGC-1α to prevent senescence, by stimulating p53 and DDR. However, the extent of the stress can exceed the potential of SIRT1/PGC1α/p53 action, and result in senescence or/and a programmed cell death. The type of such death in RPE cells in AMD is a matter of debate [95].

Saint-Geniez et al. showed that PGC-1α was expressed in many kinds of retinal cells, also during postnatal development [96]. These authors also showed that the expression of PGC-1α dramatically increased in an oxygen-induced retinopathy model, suggesting that this protein drives a pathological neovascularization typical for wet AMD. Although it is likely that the main effect of PGC-1α on neovascularization is mediated in Müller cells, which are the main source of VEGF, other cells, including RPE cells, could be also involved [97]. Therefore, expression of PGC-1α is necessary for normal development of vessels in the retina, but its overexpression can result in pathological neovascularization, observed in wet AMD. On the other hand, PGC-1α protects against oxidative stress, a major factor of AMD pathogenesis and mitochondrial damage, often observed in AMD retinas. Therefore, strict control of the PGC-1α expression in retinal cells is required to protect them against detrimental factors and prevent pathological neovascularization.

Mimura et al. presented conclusive arguments that SIRT1 could play an important protective role against age-related diseases, including AMD [98]. These protective effects of SIRT1 can be exerted through PGC-1α activation.

**Figure 6:**
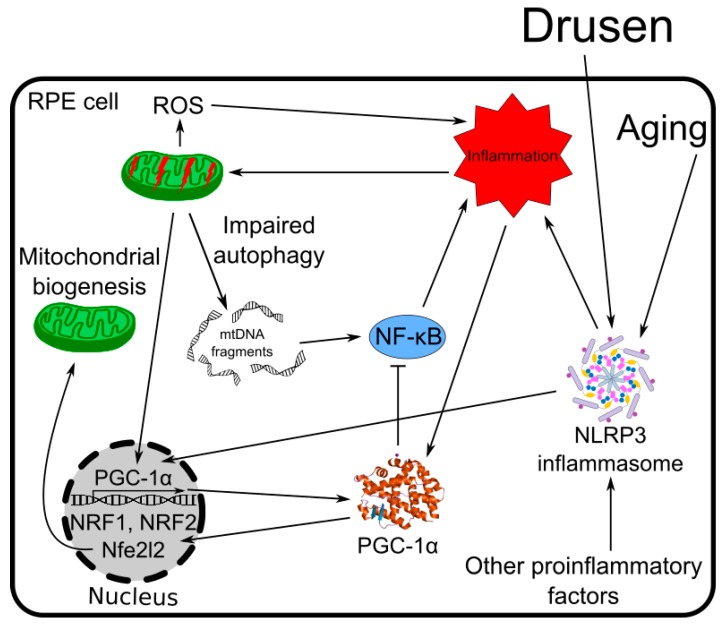
PGC-1α influences chronic inflammation associated with AMD. Degenerative changes in RPE cells observed in AMD and age-related changes in the immune system induce activation of the NLRP3 inflammasome and chronic inflammation in RPE cells. Inflammation affects mitochondria and induces their biogenesis, which is stimulated by PGC-1α with the involvement of many factors, including NRF-1 and Nfe212. Impaired mitochondria release mtDNA in cytosol, inducing NF-κB, a complex controlling inflammation, whose activity is inhibited by PGC-1α. Full names of proteins are in the main text.

Chronic inflammation is associated with AMD, and it can be induced by many factors resulting from degenerative changes in RPE cells [99] (Figure 6). Inflammation is also associated with age-related changes in the immune system. Inflammatory cellular stress affects mitochondria and mtQC evokes PGC-1α-dependent mitochondrial biogenesis [32]. Among many transcription factors regulated by PGC-1α, those directly related to mitochondrial biogenesis in inflammation are NRF-1 and Nfe2l2 [32,100,101,102]. The activation of the NLRP3 (NACHT, LRR, and PYD domain-containing protein 3) inflammasome can be critical in AMD pathogenesis [99]. Mitochondrial dysfunction evoked by AMD-related factors and inflammation induces other inflammatory reactions, leading to NLRP3 assembly [28]. This process is enforced by impaired mitophagy or, in general, autophagy, as it results in a diminished cellular clearance of waste produced by dysfunctional mitochondria, including mtDNA in cytosol, which in turn, upregulates NF-κB (nuclear factor kappa-light-chain-enhancer of activated B cells), which is a master of the inflammatory response [19]. Therefore, inflammation results in mitochondrial dysfunction, which leads to inflammasome activation, a critical event in AMD pathogenesis, and stimulates PGC-1α to co-activate transcription of several genes important for mtQC. However, PGC-1α can be directly involved in the reduction of inflammatory reaction by decreasing the activity of NF-κB, as shown in skeletal muscle cells [103].

Golestaneh et al. showed that RPE cells obtained from AMD patients by dedifferentiation of skin, and RPE cells induced into pluripotent stem cells (iPSCs) and their differentiation into RPE cells, displayed a repressed PGC-1α, as compared with RPE cells obtained from iPSCs from normal donors [104]. These authors showed that AMD RPE cells had a lower expression of SIRT1, which immediately suggests a possible mechanism of observed repression of PGC-1α. Additionally, inactivation of AMPK, the other protein responsible for PGC-1α activation, was suggested in that experiment, and altogether, the authors concluded that the repression of the AMPK/SIRT1/PGC-1α pathway could disturb mitochondrial biogenesis, resulting in overproduction of ROS, RPE cell damage, and finally, retinal degeneration typical for AMD.

SanGiovanni et al. postulated that variation in the PPARGC1A gene can influence the process of neovascularization in wet AMD [105]. They found that polymorphisms of that gene, located in its coding region and 3′ UTR, were independently associated with the occurrence of wet AMD. The study of gene–gene interaction revealed interactions of PPARC1A polymorphisms with variants of AMD-related genes, including the complement and VEGF-signaling.

Ferrington et al. using primary RPE cells from 14–16 AMD patients and 7–9 controls reported a significant, over 2-fold increase in the PGC-1α expression in AMD patients [59]. They also observed a reduced oxidative phosphorylation in AMD cells, and a decrease in PGC-1α expression in RPE cells challenged with hydrogen peroxide in both AMD patients and controls, but at 6 h after hydrogen peroxide exposure, PGC-1α expression was higher in the AMD group. In general, a reduced bioenergetic profile was observed in RPE cells from AMD donors, but despite this, these cells displayed a higher resistance to mitochondrial and glycolytic oxidative inactivation, and death induced by oxidative stress and PGC-1α might contribute to this increased resistance.

## 7. Conclusions and Perspectives

We believe that PGC-1α plays a key role in AMD pathophysiology and represents potential as a target in the prevention and treatment of this disease. Further studies are needed to establish all functions of PGC-1α in the retina, but although most experiments on the activity of PGC-1α were performed in muscle cells, they strongly suggest a central role of this protein in the pathogenesis of age-related diseases.

As mentioned, Xiong et al. showed that PGC-1α could protect against age-related chronic diseases through modulation of DDR [91]. It seems that the SIRT1/PGC-1α pathway and its involvement in the regulation of circadian rhythm is especially important in the protective role of PGC-1α against age-related diseases, especially metabolic disorders [106,107,108,109].

To experimentally proof an important role of PGC-1α in AMD, retina-specific knockouts are needed to determine functioning of AMD-related structures in normal state and conditions related to AMD, including aging and oxidative stress, leading to cellular senescence, degeneration, and death. However, it should be taken into account that PGC-1α can be, at least in part, replaced by its analogue, PGC-1β, so apart from PGC-1α knockout, PGC-1β and PGC-1α/PGC-1β knockouts can also be used.

As mentioned, telomere shortening and dysfunction occurs in both replicative and stress-induced senescence, and evokes a DDR pathway with upregulation of p53. Such dysfunction can be associated with mitochondrial impairment and downregulation of the PGC-1α network, senescence, and apoptosis [110]. An association between PGC-1α and the activity of telomerase, an enzyme which can ameliorate telomere dysfunction, has been reported, but in general, telomerase is not active in normal cells [76]. However, senescent or degenerative RPE cells are no longer normal, and this issue, i.e., telomere maintenance in AMD, should be addressed, especially in the context of the p53-PGC-1α axis.

Inflammation results in mitochondrial dysfunction, which leads to inflammasome activation, a critical event in AMD pathogenesis. Mitochondrial dysfunction usually affects the electron transport chain and results in ROS overproduction. These ROS overlap with inflammation, evoking further inflammatory reactions and further mitochondrial dysfunctions activating mtQC mechanisms with an important involvement of PGC-1α. In these events, PGC-1α can also transactivate genes of antioxidant defense, including SOD2, which is essential in clearing excess of mitochondrial ROS.

As it was suggested that senescence could result in impaired mitophagy, leading to SAMD, and this can be linked with the aging processes, it is important to establish the role of PGC-1α in the regulation of mitophagy or general autophagy. Mitophagy can be a critical element in the association between SAMD and senescence, and impaired autophagy is closely linked to AMD pathogenesis [50,66,111].

As we have mentioned several times, chronic inflammation and activation of the NLRP3 inflammasome could play a role in AMD pathogenesis. Recently, Kosmidou et al. questioned the expression of NLRP3 in RPE cells [112]. However, this does not exclude the inflammation/inflammasome activation pathway in AMD pathogenesis, as Bain et al. showed NALP1 (NACHT, LRR, and PYD domains-containing protein 1) and NALP3 inflammasome activation in human RPE cells [113].

**Figure 7:**
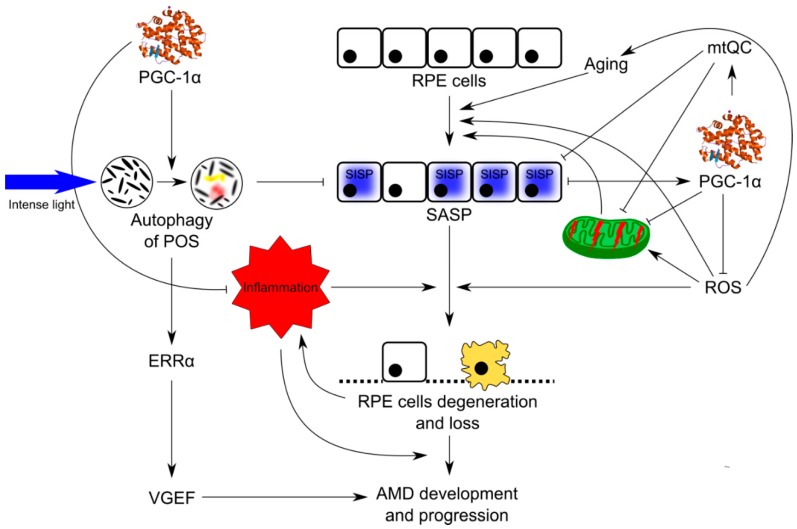
Possible involvement of PGC-1α in AMD pathogenesis. Oxidative stress, resulting in ROS overproduction, evokes stress-induced premature senescence (SIPS) in aging RPE cells, leading to their degeneration and death observed in AMD. PGC-1α negatively regulates oxidative stress and senescence. SIPS and ROS excess result in disruption of mitochondria, inducing mechanisms of mitochondrial quality control (mtQC) and mitochondrial biogenesis with the involvement of PGC-1α. Mitophagy/autophagy seems to be especially important in that context. Processing of photoreceptor outer segments (POS) with involvement of PGC-1α and modulation of inflammation by this protein are other mechanisms of its protective action against AMD. Stimulation with intense light enhanced POS phagocytosis by RPE cells, which induced the activation of the PGC-1α/ERRα pathway, which in turn upregulated VEGF.

Many mechanisms can underline the role of PGC-1α in AMD pathogenesis, and multi-pathway regulation of senescence can be especially important in this regard (Figure 7). Further studies on the detailed role of PGC-1α in the retina are needed, especially in retinal DDR, autophagy, and mtQC in normal and pathogenic conditions, to assess the potential role of that protein in AMD prevention and therapy.

In this review, we have focused on the protective action of PGC-1α in the retina, and tried to present possible mechanisms underlying that action. However, as we mentioned in the previous section, PGC-1α was reported to be involved in the upregulation of VEGF, which is crucial for wet AMD, and is targeted in the therapy of this disease [85]. However, these results do not suggest a detrimental role of PGC-1α in AMD pathogenesis, but point at this protein as an element of a signaling pathway which can be activated in specific, non-physiological conditions. On the other hand, we have concentrated on the degeneration of RPE cells, which is not directly related to choroidal neovascularization typical for wet AMD. Furthermore, although wet AMD is still AMD, some consider dry AMD and wet AMD as two separate diseases. Nevertheless, PGC-1α cannot be seen as a protein, which shows only protective action against pathological changes in the retina occurring during AMD pathogenesis. Instead, its regulation in AMD prevention and therapy also should consider PGC-1α silencing by its specific inhibitors. However, PGC-1α displays many activities in many tissues and organs, and many tissue-specific inhibitors can be applied. However, even though we limit our consideration to the retina, inhibition should concern only specific functions of PGC-1α. The problem of specificity of the inhibition of PGC-1α activity in the retina is not known and should be addressed in future research. However, it is justified to state that PGC-1α impairment in the retina may result in AMD or AMD-like state. Moreover, it can be speculated that autophagy and mitochondrial quality control can be involved, and this speculation has been recently supported by experiments of Zhang et al. [114]. These authors showed that mice with the deletion of one copy of the PGC-1α gene and fed with a fat-rich diet showed AMD-like phenotype in RPE, including diminished mitochondrial activity and autophagic flux. Therefore, such mice with a heterozygous PGC-1α deletion, fed with high fat diet, can be considered as an animal model to study AMD pathogenesis.
